# Can ionic diffusion have an effect on extracellular potentials?

**DOI:** 10.1186/1471-2202-16-S1-P68

**Published:** 2015-12-18

**Authors:** Geir Halnes, Tuomo Mäki-Marttunen, Klas H Pettersen, Daniel Keller, Ole A Andreassen, Gaute T Einevoll

**Affiliations:** 1 Dept. of Mathematical Sciences and Technology, Norwegian University of Life Sciences, Ås, Norway; 2 NORMENT, Institute of Clinical Medicine, University of Oslo, Oslo, Norway; 3 Centre for Molecular Medicine Norway, University of Oslo, Oslo, Norway; 4 The Blue Brain Project, EPFL, Lausanne, Switzerland; 5 Department of Physics, University of Oslo, Oslo, Norway

## 

Several pathological conditions, such as hypoxia, anoxia, ischemia and spreading depression are associated with ion concentration changes in the extracellular space (ECS) [1]. Also during non-pathological conditions, endured periods of intense neural signaling may cause local ion concentration changes in the millimolar range. Changes in ion concentrations are often accompanied by a slow negative potential shift measured in the ECS [1-3], the origin of which is not properly understood. In computational neuroscience, it is common to use the simplifying assumption that diffusive currents (along concentration gradients) are negligible compared to Ohmic currents (along electrical fields). This is, for example, an underlying assumption in the cable equation used in most multi-compartmental neural models. It is also an underlying assumption in standard current source density (CSD) theory, which predicts neural current sources from recordings of extracellular potentials. However, theoretical studies have identified scenarios where large, but biologically realistic, ion concentration gradients may induce diffusive currents that are comparable in magnitude to Ohmic currents [4,5].

In the current work, we have explored possible effects that diffusive currents may have on the extracellular potential in a cortical column. To do this, we developed a hybrid simulation framework: First, we used the simulator NEURON (and an available multicompartmental neural model with realistic morphology [6]) to simulate the transmembrane ionic output from a small population of cortical neurons driven by realistic synaptic input. Next, we used a separate scheme to predict variations in extracellular potentials and ion concentrations. The scheme was based on the Nernst-Planck equations for electrodiffusion and the constraint of electroneutrality, and represents a novel, efficient and generally applicable method for simulating extracellular dynamics surrounding multicompartmental neural models or networks of such (e.g., the Blue Brain simulator).

Our key findings were: (i) Morphology-dependent distributions of transmembrane current sources/sinks induced sustained extracellular potentials. Spatial variations of the ECS potential were of the order of a few mV across the depth of the cortical columns, which is similar to experimentally observed sustained potential profiles [2,3]. (ii) Long periods of neuronal output (simulations were run for 80 s) could change local ECS ion concentrations by several millimolars. (iii) For large, but realistic, concentration gradients in the ECS, diffusive currents were of the same magnitude as Ohmic currents. (iv) Diffusive currents could have a significant impact on the sustained extracellular potentials, and could be a possible explanation to the observed relationship between ionic shifts and voltage drops in the ECS [1-3]. (v) Variation in concentrations and diffusive currents took place at the time scale of several seconds, and had little impact on the frequencies that are of predominant interest in standard CSD-analysis.
